# A Treatise on the Diseases of the Ear

**Published:** 1895-03

**Authors:** 


### Reviews of Books.

*A Treatise on the Diseases of the Ear.* By T. MARK HOVELL. Pp. xxiv., 720. London: J. & A. CHURCHILL. 1894.

This is one of the most complete works on the subject of Ear Diseases in the English language, and is brought so well up to date that it will largely take the place of the translations of German works, upon which we have hitherto had to rely for anything like a full account of the subjects here treated. The book contains 122 illustrations, and although no less than half of these are of instruments used in aural surgery, yet we are not among those who would omit these, as they greatly facilitate the written descriptions of the various operations and manipulations.

The author begins with a thoroughly good account of the anatomy and physiology of the ear. This account is not a mere transcript of the usual hackneyed chapter found in many English text-books, but contains a good description of the most modern views of the structure and functions of the organ of hearing. Other chapters follow, dealing with the examination of patients suffering from the various forms of deafness, and here the valuable experience of the author enables him to deal lucidly with the manipulations and experiments necessary to form an accurate diagnosis.

Diseases of the different parts of the auditory apparatus are next described, and the important subject of treatment is fully considered. Among the best parts of the book is that dealing with the intra-cranial complications of ear diseases, which are so often neglected by works on aural surgery and relegated to works on general surgery.

The author quotes largely from Schwartze, Politzer, Barker, Lane, and other well-known workers in this department; but he perhaps hardly does justice to the good work done by Stacke (although he certainly gives a description of an operation by him for caries of the temporal bone), as he omits without any mention the operation known as "Stacke's operation" for the permanent relief of obstinate cases of antral suppuration.

Deaf-mutism is very briefly dealt with in a short chapter of seven pages, and the author is wise in not attempting to deal fully with such an important subject as this in a work intended for the practical surgeon. Upon the whole, we consider that the book is good as well in its omissions as in its contents, and that it will take high rank as a standard English text-book.

